# Ceftriaxone-Resistant Gonorrhea — China, 2022

**DOI:** 10.15585/mmwr.mm7312a2

**Published:** 2024-03-28

**Authors:** Xiaoyu Zhu, Yue Xi, Xiangdong Gong, Shaochun Chen

**Affiliations:** ^1^Hospital for Skin Diseases, Institute of Dermatology, Chinese Academy of Medical Sciences & Peking Union Medical College, Nanjing, China; ^2^National Center for AIDS/STD Control and Prevention, Chinese CDC, Nanjing, China; ^3^School of Public Health, Nanjing Medical University, Nanjing, China.

**Summary**

**What is already known about this topic?**

Gonorrhea is the fourth most reported notifiable infectious disease in China. Emergence and spread of ceftriaxone-resistant clones of *Neisseria gonorrhoeae* in China have posed a challenge to gonorrhea treatment.

**What is added by this report?**

During 2017−2022, the prevalence of antibiotic-resistant strains of *N. gonorrhoeae* increased in China, with resistance to ceftriaxone, the first-line treatment for gonorrhea, approximately tripling. Resistance varied by geographic region. Gonorrhea strains were resistant to other antibiotics at prevalences up to 97.6%, varying by antibiotic type.

**What are the implications for public health practice?**

Effective diagnosis and treatment are essential to protect the health of infected persons and prevent ongoing transmission of antibiotic-resistant gonorrhea. Identifying reasons for the spread of ceftriaxone-resistant *N. gonorrhoeae* in China could guide strategies, such as antibiotic stewardship, to curb the spread of resistant strains.

## Abstract

Gonorrhea is a widespread sexually transmitted infection; in 2022, China reported 96,313 cases of gonorrhea, making it the fourth most common notifiable infectious disease in the country after viral hepatitis, pulmonary tuberculosis, and syphilis. The rise in prevalence in antimicrobial-resistant strains, particularly the international spread of ceftriaxone-resistant clones, poses a formidable challenge to gonorrhea control. The China Gonococcal Resistance Surveillance Program (China-GRSP), established in 1987 and covering 19 of 34 provincial-level administrative units, continuously monitors gonococcal antimicrobial resistance. In 2022, 13 China-GRSP sentinel sites collected 2,804 gonococcal isolates, representing 2.9% of all cases reported in China, and 4.1% of cases reported in the 13 participating provinces. The prevalence of *Neisseria* *gonorrhoeae* resistance to ceftriaxone was 8.1%, approximately three times the 2017 rate of 2.9%; five provinces reported >10% ceftriaxone resistance. Resistance prevalences to cefixime, azithromycin, tetracycline, penicillin, and ciprofloxacin were 16.0%, 16.9%, 77.1%, 77.8%, and 97.6%, respectively. Only one case of spectinomycin resistance was reported. These data highlight a substantial increase in ceftriaxone resistance from 2017 to 2022. Effective diagnosis and treatment and appropriate management of sex partners are essential to protect the health of infected persons and prevent ongoing transmission of gonorrhea, including transmission of resistant strains. Identifying reasons for the spread of ceftriaxone-resistant *N.* *gonorrhoeae* in China could guide strategies, such as antibiotic stewardship, to mitigate the rising resistance rate and curb the spread of resistant strains.

## Introduction

Gonorrhea, a sexually transmitted bacterial infection caused by *Neisseria gonorrhoeae*, remains prevalent worldwide. The World Health Organization (WHO) estimated that approximately 82.4 million new gonorrhea cases were diagnosed among persons aged 15–49 years in 2020.^†^ In China, a total of 96,313 gonorrhea cases were reported in 2022, representing a rate of 6.83 reported cases per 100,000 population, the fourth highest among class A and class B notifiable infectious diseases^§^ in the country,^¶^ after viral hepatitis, pulmonary tuberculosis, and syphilis. In the United States, in 2022, a total of 648,056 cases of gonorrhea were reported.**

In recent years, gonococcal resistance to multiple antibiotics has emerged (*1*). Ceftriaxone is recommended as the first-line treatment option for gonorrhea in China (single dose of 1 g, administered intramuscularly)^††^ as well as in the United States (single dose of 500 mg for persons weighing <150 kg, administered intramuscularly).^§§^ However, the emergence of ceftriaxone-resistant strains, particularly the ceftriaxone-resistant clone FC428 (*2*), has been identified worldwide. First identified in Beijing in 2016 (*3*), this resistant clone has become widely disseminated across various regions of China, with its proportion among all resistant clones steadily increasing since 2016, highlighting the challenge associated with addressing gonococcal resistance (*4*).

The China Gonococcal Resistance Surveillance Program (China-GRSP), established in 1987, monitors gonococcal resistance to azithromycin, cefixime, ceftriaxone, ciprofloxacin, penicillin, spectinomycin, and tetracycline in China (*5*). This report describes gonococcal resistance surveillance data from China for 2022, the most recent year for which data are available.

## Methods

In 2022, China-GRSP conducted gonococcal resistance surveillance across 13 of the 19 provinces (among 34 national province-level administrative jurisdictions) that participate in the program, within six of seven regions of China (Supplementary Figure, https://stacks.cdc.gov/view/cdc/150923). *N. gonorrhoeae* isolates obtained from urethral (from males) or endocervical (from females) swab specimens were collected from the 2,804 identified cases included in the surveillance program from consecutively evaluated patients throughout the year. Consecutive evaluation involved specimen collection at each sentinel site from January through December, with some sites having larger sample sizes and sampling limitations that might result in data collection ending as early as September. Specimens were cultured on selective gonococcal culture media, and *N. gonorrhoeae* (an oxidase-positive, gram-negative diplococcus) was identified by microscopic examination of Gram-stained material, detection of a rapid oxidase reaction, and carbohydrate utilization test results.^¶¶^ The susceptibility of isolates to azithromycin, cefixime, ceftriaxone, ciprofloxacin, penicillin, spectinomycin, and tetracycline was determined using the agar dilution method. Antibiotic resistance breakpoints were applied based on the European Committee on Antimicrobial Susceptibility Testing criteria,*** except for azithromycin, for which WHO criteria were used. WHO *N. gonorrhoeae* reference strains were used for quality assurance. The determination of antibiotic resistance was based on the minimum inhibitory concentration (MIC) values obtained through agar dilution. The antibiotic resistance breakpoints were as follows: azithromycin MIC >0.5 mg/L, cefixime MIC >0.125 mg/L, ceftriaxone MIC >0.125 mg/L, ciprofloxacin MIC >0.06 mg/L, penicillin MIC >1 mg/L, spectinomycin MIC >64 mg/L, and tetracycline MIC >1 mg/L. Resistance rate was expressed as the percentage of resistant isolates among the total number of isolates. This activity was reviewed and approved by the Medical Ethics Committee at the Institute of Dermatology, Chinese Academy of Medical Sciences & Peking Union Medical College, and the National Center for AIDS/STD Control and Prevention in China.

## Results

In 2022, a total of 2,804 isolates (4.1% of 68,217 gonococcal infection cases) from 13 provinces in China were tested for antimicrobial susceptibility. The largest numbers of cases were reported in Guangdong (22,171) and Zhejiang (13,460) provinces. Rates of reported cases ranged from 2.13 to 20.58 per 100,000 population, with highest rates reported in Zhejiang, Guangdong, Yunnan, Hainan, and Guangxi provinces (Table 1).

**TABLE 1:** Reported cases and rates of gonorrhea and proportion of isolates available for antimicrobial susceptibility tests, by province — 13 Gonococcal Resistance Surveillance Program sentinel sites,* China, 2022

Province	Population	No. of reported gonorrhea cases	Rate^†^	No. of isolates tested for antimicrobial susceptibility (%)
Chongqing	32,119,942	2,498	7.78	66 (2.6)
Guangdong	126,840,013	22,171	17.48	751 (3.4)
Guangxi	50,369,886	6,162	12.23	719 (11.7)
Hainan	10,199,964	1,466	14.37	57 (3.9)
Hunan	66,220,222	3,016	4.55	66 (2.2)
Jiangsu	85,050,277	5,354	6.30	248 (4.6)
Shanghai	24,889,864	1,339	5.38	111 (8.3)
Shanxi	39,539,596	1,405	3.55	65 (4.6)
Sichuan	83,721,532	3,235	3.86	120 (3.7)
Tianjin	13,730,084	293	2.13	53 (18.1)
Xinjiang	25,889,690	905	3.50	27 (3.0)
Yunnan	46,899,911	6,913	14.74	146 (2.1)
Zhejiang	65,400,126	13,460	20.58	375 (2.8)

* Data from 13 of 19 provincial sentinel surveillance sites were included in the analysis; only 2,804 isolates were tested for antimicrobial susceptibility, accounting for 4.1% of all reported cases in the 13 participating provinces.

^†^ Per 100,000 population.

Percentages of isolates tested by province ranged from 2.1% (Yunnan) to 18.1% (Tianjin). Among isolates submitted, resistance was identified to ciprofloxacin (97.6%), penicillin (77.8%), tetracycline (77.1%), azithromycin (16.9%), cefixime (16.0%), and ceftriaxone (8.1%) (Table 2). Only one isolate was resistant to spectinomycin. Among 2,804 isolates, those from 18 cases were identified as resistant to all antibiotics except spectinomycin.

**TABLE 2:** Resistance of gonococcal isolates to ciprofloxacin, penicillin, tetracycline, azithromycin, cefixime, ceftriaxone, and spectinomycin — 13 Gonococcal Resistance Surveillance Program sentinel sites,* China, 2022

Province	Antibiotic/MIC, no. of resistant isolates (%)						
	Ciprofloxacin/>0.06 mg/L^†^	Penicillin/>1 mg/L^†^	Tetracycline/>1 mg/L^†^	Azithromycin/>0.5 mg/L^†^	Cefixime/>0.125 mg/L^†^	Ceftriaxone/>0.125 mg/L^†^	Spectinomycin/>64 mg/L^†^
Chongqing	64 (97.0)	43 (65.2)	28 (42.4)	14 (21.2)	16 (24.2)	9 (13.6)	0 (—)
Guangdong	741 (98.7)	604 (80.4)	509 (67.8)	161 (21.4)	172 (22.9)	66 (8.8)	0 (—)
Guangxi	714 (99.3)	538 (74.8)	631 (87.8)	115 (16.0)	65 (9.0)	45 (7.6)	1 (0.1)
Hainan	53 (93.0)	19 (33.3)	25 (43.9)	2 (3.5)	1 (1.8)	1 (1.8)	0 (—)
Hunan	66 (100.0)	59 (89.4)	26 (39.4)	25 (38.0)	10 (15.2)	2 (3.0)	0 (—)
Jiangsu	209 (84.3)	184 (74.2)	197 (79.4)	32 (12.9)	55 (28.2)	24 (12.3)	0 (—)
Shanghai	111 (100.0)	109 (98.2)	60 (54.1)	42 (37.8)	14 (12.6)	0 (—)	0 (—)
Shanxi	65 (100.0)	54 (83.0)	49 (75.4)	0 (—)	6 (9.2)	4 (6.2)	0 (—)
Sichuan	120 (100.0)	86 (71.7)	101 (84.2)	11 (9.2)	44 (36.7)	30 (25.0)	0 (—)
Tianjin	53 (100.0)	47 (88.7)	15 (28.3)	12 (22.6)	19 (35.9)	14 (26.4)	0 (—)
Xinjiang	21 (77.8)	19 (70.4)	27 (100.0)	6 (22.2)	7 (25.9)	7 (25.9)	0 (—)
Yunnan	146 (100.0)	130 (89.0)	139 (95.2)	25 (17.1)	12 (8.2)	10 (6.9)	0 (—)
Zhejiang	374 (99.7)	289 (77.1)	356 (94.9)	28 (7.5)	20 (5.3)	10 (2.8)	0 (—)
**Total**	**2,737 (97.6)**	**2,181 (77.8)**	**2,163 (77.1)**	**473 (16.9)**	**441 (16.0)**	**222 (8.1)**	**1 (<1)**

**Abbreviation:** MIC = minimum inhibitory concentration.

* Data from 13 of 19 provincial sentinel surveillance sites were included in the analysis.

**^†^** Concentrations listed are the MIC thresholds used to categorize resistant isolates.

Antibiotic resistance rates differed among provinces. Whereas ceftriaxone resistance detected in most sentinel sites was ≤5% during the past decade, in 2022, five provinces (Chongqing, Jiangsu, Sichuan, Tianjin, and Xinjiang) reported >10% ceftriaxone resistance, with rates in Sichuan, Tianjin, and Xinjiang surpassing 20%; only Hainan, Hunan, Shanghai, and Zhejiang reported ≤5% ceftriaxone resistance (Figure). Among other antibiotics, overall resistance to cefixime was 16.0%, with rates in Jiangsu, Sichuan, Tianjin, and Xinjiang exceeding 25%. Azithromycin resistance was >35% in Hunan and Shanghai and >20% in Chongqing, Guangdong, Tianjin, and Xinjiang. Resistance to ciprofloxacin remained consistently high nationwide (97.6%), with Hunan, Shaanxi, Shanghai, Sichuan, Tianjin, and Yunnan reaching 100%. Overall resistance to tetracycline was 77.1%, ranging from 28.3% in Tianjin to 100% in Xinjiang. Penicillin resistance was 77.8% nationwide and was >70% in most provinces; the highest penicillin resistance rate (98.2%) was reported by Shanghai province.

**FIGURE:**
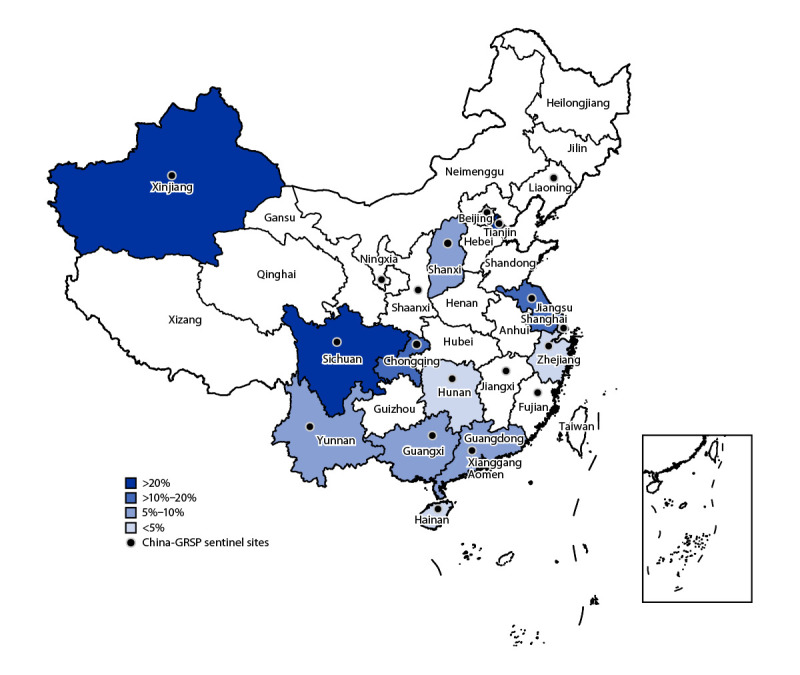
Reported rates of ceftriaxone resistance — 13 Gonococcal Resistance Surveillance Program sentinel sites,* China, 2022 **Abbreviation:** GRSP = Gonococcal Resistance Surveillance Program. * Data from 13 of 19 provincial sentinel surveillance sites were included in the analysis.

## Discussion

The prevalence of ceftriaxone resistance among gonococcal isolates in China nearly tripled since 2017, increasing from 2.9% to 8.1% in 2022; this rate is relatively high compared with that in other countries (*1*). For example, in 2022, the percentage of strains with reduced susceptibility to ceftriaxone (MIC >0.03 mg/L) in the United Kingdom was 0.21%.^†††^ According to the U.S. CDC’s Gonococcal Isolate Surveillance Project report, the prevalence of isolates exhibiting elevated ceftriaxone MICs (≥0.125 *μ*g/mL) fluctuated at approximately 0.2% during 2016−2020.^§§§^ In Canada, prevalence of decreased susceptibility to ceftriaxone has remained relatively stable, at approximately 0.6% during 2017–2021 (*6*). 

These findings underscore the urgent need for a comprehensive approach to address antibiotic-resistant *N.* *gonorrhoeae* in China, including identifying factors contributing to this high resistance rate, especially in provinces where the percentage of gonococcal isolates resistant to ceftriaxone is >10%. Factors that could contribute to ceftriaxone resistance include spread of the ceftriaxone-resistant FC428 strain, gaps in gonorrhea screening, treatment, and partner management, and nonrecommended prescribing or use of antibiotics (although antibiotics are only available by prescription in China). Understanding these factors is crucial to guiding the development and implementation of targeted interventions and preventive measures. The preliminary investigation revealed that the widespread dissemination of ceftriaxone-resistant FC428 clones might be the underlying reason for the high resistance rate in China (*3*,*4*,*7*), although whole-genome sequencing of isolates collected in 2022 is ongoing. These resistant clones have spread internationally (*8*–*10*), and collaborative cross-border efforts will be essential to monitoring and mitigating its further spread. These findings also reinforce the pivotal role of programs such as the China-GRSP in the ongoing monitoring and adapting of strategies to address evolving resistance patterns. The observed resistance rates for other antibiotics emphasize the complex landscape of gonococcal antimicrobial resistance, further highlighting the urgent need to develop alternative treatment strategies, including vaccines to counter this growing threat.^¶¶¶^

### Limitations

The findings in this report are subject to at least four limitations. First, relying on reported cases of gonorrhea might underestimate the actual incidence, because asymptomatic cases or those among patients not seeking medical attention might go unrecorded. Second, in 2022, China-GRSP only covered one third of the country, and fewer than 3% of isolates were available for testing, leading to potential bias, and results might not be representative of the entire country. Third, this analysis focused on antimicrobial resistance rates and did not address broader sociodemographic factors influencing gonorrhea transmission. Finally, the lack of detailed patient information hampers the identification of specific risk factors contributing to the observed resistance patterns. Future research should address these limitations for a more nuanced understanding of *N.* *gonorrhoeae* epidemiology in China.

### Implications for Public Health Practice

The increasing prevalence of ceftriaxone resistance in *N.* *gonorrhoeae* in China highlights a pressing public health concern. Effective diagnosis and treatment and appropriate management of sex partners are essential to protect the health of infected persons and prevent ongoing transmission of gonorrhea, including transmission of resistant strains. Public health practitioners should prioritize assessment of screening practices, particularly in regions with higher reported rates of gonorrhea cases and resistance rates. Understanding the factors that could contribute to the spread of resistance, such the nonrecommended use of antimicrobials, is also crucial to guide prevention efforts. Collaborative efforts and ongoing surveillance to monitor the international spread of resistant strains, as exemplified by programs like China-GRSP, are vital for a global response. International collaboration and information sharing are critical to prevent the further cross-border spread of resistant strains and to identify alternative treatment options for gonorrhea. Given the identified limitations, future research should aim to broaden surveillance coverage, incorporate detailed patient information, and conduct a comprehensive analysis of sociodemographic factors. These efforts could improve understanding of gonococcal infections and antibiotic resistance in China. The findings underscore the dynamic nature of this public health issue, emphasizing the ongoing need for adaptive and collaborative approaches to address the growing threat of antibiotic-resistant *N.* *gonorrhoeae* effectively.

## Footnotes

* These authors contributed equally to this report.

†
https://www.who.int/news-room/fact-sheets/detail/gonorrhoea-(neisseria-gonorrhoeae-infection)

§ Two class A and 28 class B notifiable infectious diseases are recognized in China; class A diseases include cholera and plague, and class B diseases include those associated with a high risk for outbreaks or that are likely to result in rapid spread once an outbreak occurs, such as AIDS, gonorrhea, measles, syphilis, and tuberculosis.

¶ http://www.nhc.gov.cn/guihuaxxs/s3585u/202309/6707c48f2a2b420fbfb739c393fcca92.shtml; data on rates of reported cases of COVID-19 were not available; therefore, SARS-CoV-2 infections were not included in these statistics.

**
https://www.cdc.gov/std/statistics/2022/overview.htm#Gonorrhea

††
https://doi.org/10.1097/JD9.0000000000000072

§§
https://www.cdc.gov/std/treatment-guidelines/gonorrhea-adults.htm

¶¶
https://www.who.int/publications/i/item/9789241505840

***
www.eucast.org/clinical_breakpoints/

†††
https://www.gov.uk/government/publications/gonococcal-resistance-to-antimicrobials-surveillance-programme-grasp-report/grasp-report-data-to-june-2023

§§§
https://www.cdc.gov/std/statistics/gisp-profiles/default.htm

¶¶¶
https://www.who.int/publications-detail-redirect/9789240039827
